# Response Surface Methodology Approach for Predicting Convective/Infrared Drying, Quality, Bioactive and Vitamin C Characteristics of Pumpkin Slices

**DOI:** 10.3390/foods12051114

**Published:** 2023-03-06

**Authors:** Fatemeh Joudi-Sarighayeh, Yousef Abbaspour-Gilandeh, Mohammad Kaveh, Mariusz Szymanek, Ryszard Kulig

**Affiliations:** 1 Department of Biosystems Engineering, College of Agriculture and Natural Resources, University of Mohaghegh Ardabili, Ardabil 56199-11367, Iran; 2 Department of Petroleum Engineering, College of Engineering, Knowledge University, Erbil 44001, Iraq; 3 Department of Agricultural, Forest and Transport Machinery, University of Life Sciences in Lublin, Głęboka 28, 20-612 Lublin, Poland; 4 Department of Food Engineering and Machines, University of Life Sciences in Lublin, Głęboka 28, 20-612 Lublin, Poland

**Keywords:** drying, pumpkin, RSM, total phenolic content, shrinkage

## Abstract

In this research, a convective/infrared (CV/IR) dryer was used to dry pumpkin slices. For optimization of the drying conditions, the influence of three levels of independent variables including air temperature (40, 55, and 70 °C), air velocity (0.5, 1, and 1.5 m/s), and IR power (250, 500, and 750 W) were assessed by response surface method (RSM) through a face-centered central composite design. Analysis of variance (non-fitting factor and R^2^ value) was employed to determine the desirability of the model. Response surfaces and diagrams were also utilized to show the interactive influence of the independent variables with the response variables (drying time, energy consumption, shrinkage, total color variation, rehydration ratio, total phenol, antioxidant, and vitamin C contents). According to the results, optimal drying conditions involved a temperature of 70 °C, air velocity of 0.69 m/s, and IR power of 750 W. At the mentioned conditions, response variables of drying time, energy consumption, shrinkage, color, rehydration ratio, total phenol, antioxidant, and vitamin C contents were 72.53 min, 24.52 MJ/kg, 23%, 14.74, 4.97, 617.97 mg GA/100 g dw, 81.57%, and 4.02 mg/g dw, with a confidence level of 0.948, respectively.

## 1. Introduction

Pumpkin (*cucurbita maxima*) is one of the most important crops all around the world. This product contains highly bioactive compounds with health-boosting abilities such as polyphenols, antioxidant, carotenoids (mainly β-carotene), vitamins C and A, minerals, polysaccharides, and edible fibers [1,2]. The presence of bioactive compounds in pumpkin shows it as a functional food or ingredient for the development of a variety of innovative food products with health-promoting properties [3]. Additionally, due to its bioactive properties, carotenoids, and vitamins, pumpkin has beneficial effects on human health, including: reducing the risk of neurological, heart, and cancer diseases [4], the prevention of osteoporosis [5], and high blood pressure [6]. The high moisture content of pumpkin (90.1 ± 0.3%), however, makes it prone to microbial decay. Hence, pumpkin fruits need to be kept either frozen or dried. Pumpkin is often distributed in the form of a raw vegetable, and a processed product (frozen, dried, puréed, or pre-cooked) to enhance its stability during storage [7]. Drying can prolong the useful life of food products and play a decisive role in agricultural products by evaporating their moisture content, hence meddling with the growth of the microbial agents which grow in humid media [8]. Despite its time-consuming and energy-demanding nature, drying approaches are necessary in the production of sustainable products with minimum water contents which can cause a dramatic decline in the weight and volume of the product, hence facilitating its storage and transport [9,10,11]. The drying methods can be generally divided into traditional and industrial classes [12]. The long drying time in the traditional methods can increase the possibility of microbial growth; on the other hand, the industrial methods can maintain the quality of the foods at a desirable level while drastically shortening the drying time [13,14].

The quality of the final product is one of the main indicators of the drying process as the quality may vary during the mass and heat transfer or chemical reactions, affecting the consumer satisfaction in terms of the physical (shape, color, and texture) and nutritional (vitamins, antioxidants, and phenolic compounds) features [15]. Regarding the high latent heat of water and relatively low energy efficiency of the industrial dryers, high input energy is required for the drying process, leading to high heating costs [16]. Among the various dying methods, one capable of decreasing the energy consumption and drying time while enhancing the quality of the final product is highly welcome. A convective (hot air) dryer is a simple, conventional, and low-cost method for drying food products which suffers from a long drying time and low energy efficiency [17,18,19]. Infrared drying, on the other hand, can provide fast and uniform heat distribution through electromagnetic waves within the product, decreasing the energy consumption and drying period while enhancing the quality of final product [20,21]. A combinational use of convective and IR dryers can offer the advantages of both methods (i.e., maintaining the quality of the final product and shorter drying time) [22]. Hybrid dryers have been used for drying various products including rice by fluidized bed-IR-microwave [23], apple by microwave/CV and CV/IR [24], chrysanthemum by CV/IR dryer [25], walnut kernel by the IR-vacuum and IR-fluidized bed with microwave pretreatment [26], tomato by CV/IR and microwave-CV [27], and okra by IR-freeze-drying and microwave vacuum [28].

The response surface method (RSM) refers to a series of mathematical and statistical methods to model and analyze the problems in which the response variable is under the influence of several independent variables to optimize the response variables [29]. RSM evaluates the mutual relationships between the input (independent) variables and their impact on the response (dependent) variable. The aim of the modeling is to achieve the best system performance and promote the cost-effectiveness of the drying process through rational predictions [30]. This method has been extensively utilized in drying food products such as rough rice [31], echinacea root [32], lime [33], cumin seeds [34], and apple [35].

Prashob et al. [36] determined optimal drying conditions for drying shrimp by hot air-assisted continuous infrared drying at various IR powers, temperature, and IR lamp-product distance using RSM. Nanvakenari et al. [23] also addressed the optimization of operation conditions to dry rice using a fluidized bed-assisted hybrid IR-microwave dryer through the RSM and verified the reliability of the model based on the experiments. Drying conditions of the microwave-assisted fluidized bed dryer were optimized for red bell pepper [37]. The derived model was also experimentally verified. RSM can accurately predict SEC, color, and RR for drying ginger slices in a CV/IR dryer [38]. Dhurve et al. [39] examined the mutual effects of air temperature, air velocity, IR power, and vibration intensity on the TPC, flavonoid, and *AA* of pumpkin seeds dried by a hybrid IR-vibro fluidized bed dryer using RSM. They indicated the significant influence of the input parameters on the response values.

A review of the literature showed that no study has addressed the performance and optimization of a hybrid CV/IR dryer for drying pumpkin slices considering the air temperature, air flow velocity, and IR power. In this context, a continuous CV/IR dryer was used in this study to dry pumpkin slices in which the heat was supplied by a heater (supplier of the air temperature) and four IR lamp. To this end, the operational features such as drying time, specific energy consumption (SEC), color, shrinkage, RR, and *AA*, TPC, and VC contents were assessed. Additionally, the optimal condition of the dryer was determined by selecting the proper air temperature, air velocity, and IR power using the RSM method.

## 2. Materials and Methods

### 2.1. Sample Preparation

Pumpkins were purchased form a local market in Sardasht (west Azarbyjan, Iran). The samples were then kept in plastic bags in a refrigerator (3–5 °C) to prevent the decline in initial moisture content (MC); for the tests, the samples were put at room temperature for 2 h to reach the ambient temperature [40]. Pumpkins were cut into 4-mm slices using a cutter. The initial moisture content of the samples was measured by putting the slices at 70 °C for 24 h using an oven (Memmert, UFB 500, Schwabach, Germany). The initial moisture content was obtained 6.38 ± 0.1 on dry basis (% d.b).

### 2.2. Drying

In this research, the pumpkin samples were dried in a convective/infrared (CV/IR) dryer designed by the biosystem engineering department of Mohaghegh Ardabili University, Ardabil, Iran [22]. The utilized dryer included a drying chamber (120 × 100 × 130 cm^3^), a centrifugal fan, thermal elements (3 heating elements and IR lamps), and a control unit (encompassing blowing speed controller, thermometer of the input air, and controller of the number of on/off IR lamps). Four IR lamps, (each with the power of 250 W) were placed within and above the drying chamber. The lamps were 15 cm above the samples. Regarding the test conditions, 3 levels of IR power (maximum 3 IR lamps with power of 750 w) were used. A centrifugal lamp (1 hp/3000 rpm) was utilized to supply the input air at the beginning of dryer while a gate was installed at the top of the dryer for exhaust of air and humidity. The speed of the input air was adjusted by an invertor (LS, Seoul, Republic of Korea). The temperature of the input air was also regulated by a thermostat (Atbin, Tehran, Iran) equipped with K-type thermocouples. The weight of the samples was also measured every 5 min using a digital balance (AND GF-6000) at the resolution of 0.001. The tests were continued until the MC of the pumpkin slices reached from 6.38 (d.b) of the initial MC to 0.12 (d.b). The drying tests were carried out at three air temperatures of 40, 55, and 70 °C, three IR powers of 250, 500, and 750 W, and three airflow of 0.5, 1, and 1.5 m/s in a central composite design.

### 2.3. Moisture Content

The MC of the samples was determined by Equation (1) [27]:

(1) $$MC_{d.b}=\frac{W_{i}-W_{f}}{W_{f}}$$

### 2.4. Specific Energy Consumption

The specific energy consumption refers to the ratio of the total energy consumption for drying pumpkin slices to the water loss during the drying process. The equations used to determine the SEC of a CV/IR dryer can be found in Table 1.

### 2.5. Quality Features

#### 2.5.1. Color

The color of the dried samples was evaluated based on *L*^∗^, *a*^∗^, and *b*^∗^ parameters using a color-meter (HP-200, Guangdong, China). The color change (∆*E*) in the pumpkin samples after drying can be determined by Equation (8) [46]:

(8) $$\Delta E=\sqrt{{\left(\Delta L^{*}\right)}^{2}+{\left(\Delta a^{*}\right)}^{2}+{\left(\Delta b^{*}\right)}^{2}}$$

#### 2.5.2. Shrinkage

The shrinkage of the pumpkin samples was measured by fluid displacement (toluene) [47]. The volume variations of the samples before and after drying was measured. Then, the shrinkage was determined by Equation (9) [48]:

(9) $$S_{a}=(1-\frac{V_{t}}{V_{0}})\times 100$$

#### 2.5.3. Rehydration Ratio

Water resorption can indicate the physiochemical variations during the drying process. It could also be a measure of damage made to the sample texture. To assess rehydration ratio, 5 g of the dried sample was floated in distilled water for 1 h (100 mL, 20 °C). After removing the sample from water and drying the excessive water, the rehydration ratio was determined by Equation (10) [49]:

(10) $$RR=\frac{W_{r}}{W_{d}}$$

### 2.6. Bioactive Properties

#### 2.6.1. Antioxidant Activities

Antioxidant activities of the samples was quantified by measuring the inhibitory ability against DPPH free radicals. In this method, 2 mL of the extract was mixed with 2 mL of methanolic DPPH solution and shaken in darkness for 30 min. The absorbance of the sample was measured at 520 nm. Equation (11) shows the free radicals’ inhibition percentage [50,51]:

(11) $$AA=(1-\frac{A_{s}}{A_{c}})\times 100$$

#### 2.6.2. Total Phenol Content (TPC)

Total phenol content was measured using Folin–Ciocâlteu reagent in the form of gallic acid and expressed [52]. In this method, 0.4 mL of the extract was mixed with 3 mL of water-diluted Folin–Ciocâlteu reagent solution (1:10). After resting at room temperature for 5 min, 3 mL of sodium bicarbonate (7%) was added. The solution was kept at room temperature (22 °C) for 90 min and the absorbance of the samples was spectroscopically read at 752 nm.

### 2.7. Vitamin C

Vitamin C was measured using an HPLC device (made in Iran; Danchrom hplc model). The extract preparation involved combining 300 mg of powdered sample and 30 mL of 4.5% metaphosphoric acid solution followed by 5 min of stirring. The resulting solution was then centrifuged at 4000 rpm for 15 min and the centrifuged solution was kept in a refrigerator at 4 °C for 1 h. The injection volume was 20 µL and the column temperature was 25 °C with separation using a Eurosphere column (Eurosphere, C18, 5 × 4/6 × 250). The absorption of the sample was read at a wavelength of 245 nm, the mobile phase was in 0.01% sulfuric acid, and the flow rate was 1 mL/min.

### 2.8. Response Surface Method (RSM)

The best conditions for the production of dried pumpkin samples with a CV/IR dryer were determined using a response surface method as implemented in Design Expert 10 software. In this research, the central composite design (CCD) with three independent variables (air temperature, air velocity, and infrared power) at three levels (Table 2) was employed, and their effects were assessed on the dependent variables. In the RSM, the goal is to optimize the response variable, which is influenced by many variables. The optimal value (*y*) can be obtained by solving the regression Equation (12) [29]:

(12) $$y=\beta_{0}+\sum_{i=1}^{k}\beta_{i}x_{i}+\sum_{i}\sum_{j}\beta_{ij}x_{i}x_{j}+\sum_{i=1}^{k}\beta_{ii}x_{i}^{2}+\varepsilon$$

In the above relationship, *β*_0_, *β_i_*, *β_ii_*, and *β_ij_* are parameters related to regression coefficients, while *x_i_* and *x_j_* denote independent variables. *k* represents the number of variables, and ε shows the error. The coded levels of independent variables and experimental design along with the response of drying time, SEC, shrinkage, RR, color, and TPC, *AA*, and VC contents are presented in Table 2 and Table 3 at different levels of independent variables, respectively. The test data performed at air temperature 55 °C temperature, air velocity of 1 m/s, and IR power 500 W, were repeated 6 times (Table 3).

To achieve optimal conditions in terms of the objectives of this study, the maximum value of RR, TPC, AA, and VC, as well as minimum drying time, SEC, shrinkage, and color variations, were considered.

## 3. Results and Discussion

Table 4 shows the fitted statistical values for dependent variables (drying time, SEC, shrinkage, color, RR, and TPC, AA, and VC). According to Table 4 and the statistical values of all the responses, the value of the coefficient of determination (R^2^) was above 0.91 while the coefficient of variation (C.V.) was less than (≈7) for all models, suggesting good reproducibility of models. Moreover, with the non-significance of the misfit factor indicated that all the presented models accurately predicted the changes in the dependent variables.

### 3.1. Drying Time

**Figure 1:**
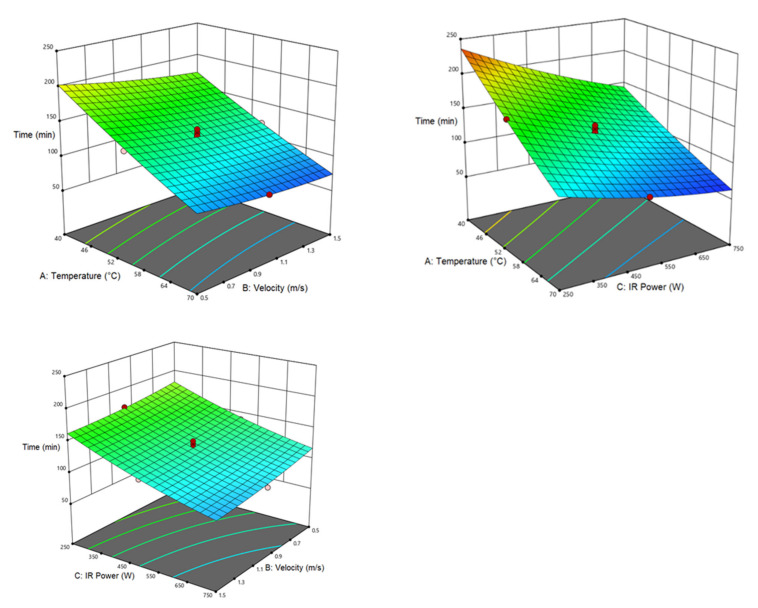
Interaction of air temperature/air velocity, air temperature/IR power, and IR power/air velocity on drying time of pumpkin.

Table 5 lists the analysis of variance for the response of drying time. Accordingly, the linear expression of air temperature, air velocity, and IR power are significant with the response of drying time (*p* < 0.0001). In addition, the second-order expression of air velocity and the interactive effect of air temperature and infrared power are also significant at the probability level of *p* < 0.0005. The second order and the interactive effect of other parameters were not significant. The longest drying time (250 min) was obtained at air temperature of 40 °C, air velocity of 0.5 m/s, and IR power of 250 W whereas the shortest drying time (60 min) was recorded at air temperature of 70 °C, air velocity of 1.5 m/s, and IR power of 750 W. Figure 1 shows the interactive effect of air temperature–air velocity and IR power–air temperature with the dependent variable of drying time. Accordingly, the drying time decreases with the elevating air temperature and IR power, due to the increase in the movement of water molecules in the product which enhanced the water evaporation rate of the samples, elevating the heat transfer flow in the samples and accelerating the evaporation. In addition, a rise in the IR power led to the rapid heating of the product, better water evaporation, and ultimately a reduced drying time [53,54,55]. Similar results were reported in drying slices of pear [56], lemon [57], and strawberry [58]. According to Figure 1, the increase in all three factors shortened the drying time. It can be said that air temperature, IR power, and air velocity show the greatest effect on reducing drying time, respectively. Simultaneous use of IR power and temperature will increase the temperature of the dryer which can increase the moisture absorption capacity due to the increase in the product–air temperature difference. On the other hand, this reduces the drying time by a faster increase in the product temperature and water evaporation [59].

### 3.2. Specific Energy Consumption

**Figure 2:**
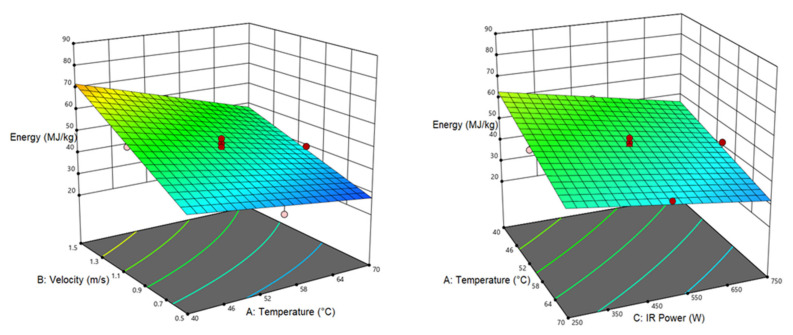

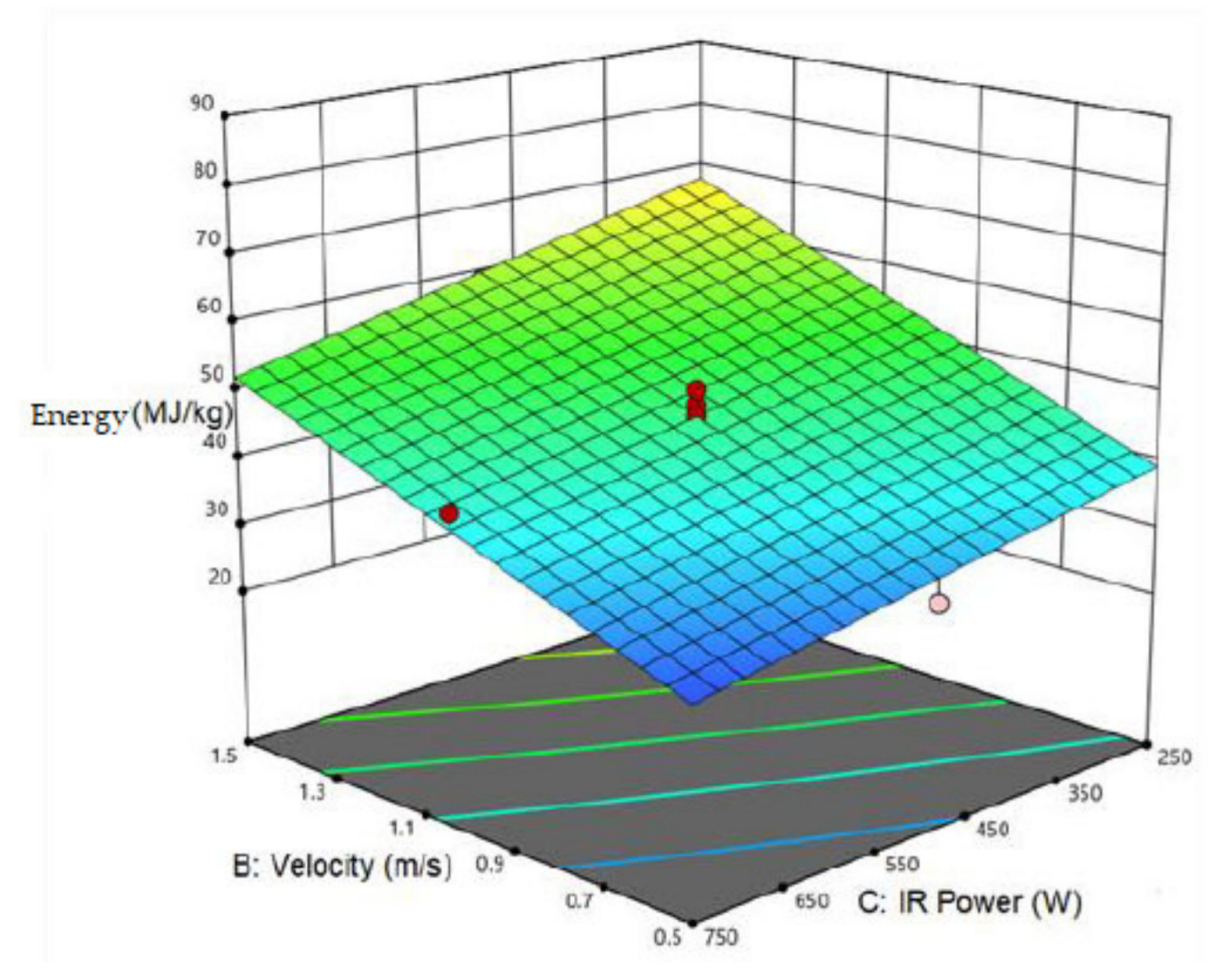
Interaction of air temperature/air velocity, air temperature/IR power, and IR power/air velocity on energy consumption of pumpkin.

Table 6 shows the analysis of variance for SEC response. Accordingly, the linear relationship between air temperature, air velocity, and IR power with the SEC variable is significant (*p* < 0.0001). The interactive air temperature–air velocity relationship with the SEC response is also significant (*p* < 0.01). Figure 2 shows the interactive effect of air temperature–air velocity on the SEC response. As seen, air temperature enhancement from 40 to 70 °C at the high air velocity (1.5 m/s) reduced the SEC with a greater slope compared to the low air velocity (0.5 m/s). However, increasing the air velocity incremented the SEC. According to Figure 2, SEC decreased with increasing IR power and air temperature. The reason could be the accelerated water evaporation at high temperature and low air velocity, which significantly reduced the drying time, hence, causing a decline in the SEC. Moreover, all energy of IR lamps is directly radiated to the sample and heats it, which decremented the energy loss [53]. Based on Figure 2, however, air velocity has a direct relationship with SEC, in other words, the increase in air velocity enhanced the SEC. At low air speeds, the SEC decreased due to the rise in the effective contact between air and pumpkin samples and increased moisture evaporation rate as a result of IR heating. The increase in the SEC with the elevation of air velocity can be attributed to the cooling of the sample surface that reduces the moisture evaporation, prolonging the drying time [60]. To improve the processing and the quality of poria cocos with vacuum and CV/IR dryers, Zhang et al. [61] concluded that the vacuum process has the highest level of SEC (6.21 MJ/kg) while CV/IR led to the lowest SEC (1.38 MJ/kg). Based on the work of Chen et al. [62], infrared heating along with hot air reduced SEC in drying carrots.

### 3.3. Quality Features

#### 3.3.1. Color

**Figure 3:**
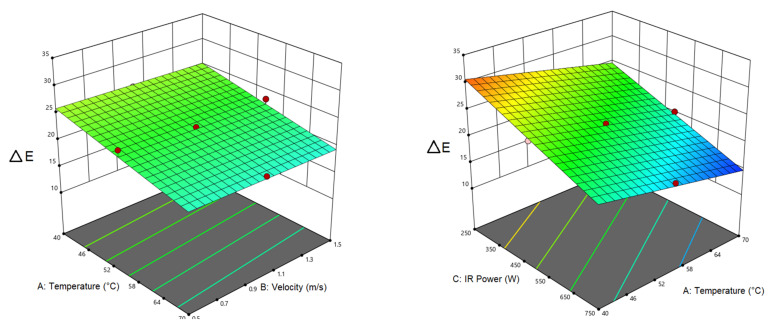
Interaction of air temperature/air velocity and air temperature/IR power on color of pumpkin.

According to Table 7, listing the analysis of variance for the color index, the linear relationship of air temperature, and IR power with the color response is significant (*p* < 0.0001). Based on Figure 3, the color changes showed a downward trend with the rising air temperature while the air velocity had no significant effect on the color, and it is not mentioned in Table 7. The greatest color change occurred at an air temperature of 40 °C and an IR power of 250 W. By raising the air temperature and infrared power, the intensity of the color decreased. The lowest color changes were recorded at 70 °C and an IR power of 750 W. One of the reasons could be the influences on the nutrients in long drying times at low temperatures. Additionally, more heat will penetrate into the samples at higher IR powers, elevating its temperature. In this way, enzymatic and non-enzymatic browning reactions, burning, and darkening of the surface of the samples were reduced. In a study on terebinth, the milder color change in the samples dried in the CV/IR method (compared to the IR dryer) is the faster drying and shorter drying time, which prevented the pigment degradation, leading to better color preservation [42].

#### 3.3.2. Shrinkage

**Figure 4:**
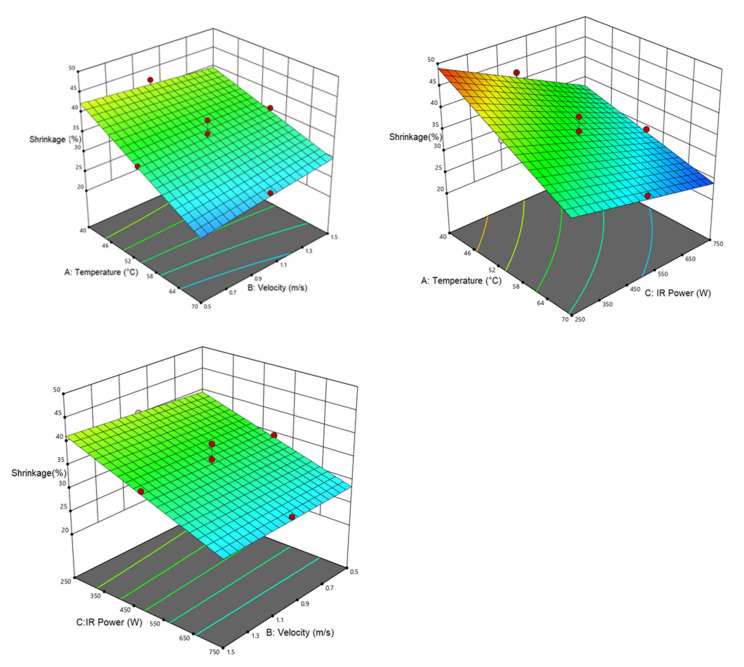
Interaction of air temperature/air velocity, air temperature/IR power, and IR power/air velocity on shrinkage of pumpkin.

Table 8 lists the analysis of variance results for shrinkage response. As seen, the linear relationship of air temperature and IR power with shrinkage is significant at (*p* < 0.0001) level. As can be observed, the air velocity had no significant influence on the shrinkage response; while the interactive air velocity–air temperature and air temperature–IR power effects were significant on the shrinkage. Figure 4 also demonstrates the interactive effects of the independent variables with the shrinkage (dependent response). As with IR power, changes in the air speed had no meaningful impact on the shrinkage; while the shrinkage showed a decline by raising the air temperature. Furthermore, temperature elevation at the low infrared power significantly decreased shrinkage compared to the high IR power. The highest shrinkage (49.95%) occurred at 40 °C and IR power of 250 W. Long-term heating leads to high moisture escape during the drying process as well as thermal stresses in the samples, hence, increasing the shrinkage [63]. El-Mesery et al. [64] dried tomato slices by a CV/IR dryer and reported a decrement in the shrinkage by increasing the IR power.

#### 3.3.3. Rehydration Ratio (RR)

**Figure 5:**
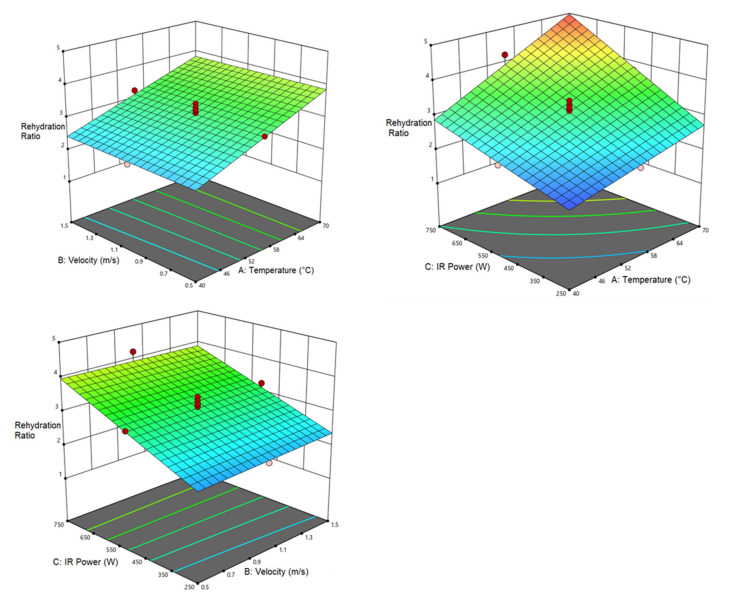
Interaction of air temperature/air velocity, air temperature/IR power, and IR power/air velocity on rehydration ratio of pumpkin.

RR is one of the major quality indicators of dry products. It can be said that better products have a higher RR. The highest RR (4.98) was obtained at an air temperature of 70 °C, air velocity of 0.5 m/s, and IR power of 750 W while the air temperature of 40 °C, air velocity of 0.5 m/s, and IR power of 250 W led to the lowest RR (1.75). Table 9 shows the results of the analysis of variance for RR response. Accordingly, the linear relationship between air temperature and IR power with RR response is significant (*p* < 0.0001). The interactive effect of air temperature–IR power is also significant (*p* < 0.0005). Other parameters had no significant influence on the resorption ratio. Figure 5 shows the mutual influence of independent variables on RR response. As seen, a rise in the air temperature enhanced the RR, while air velocity showed no effect. Moreover, RR increased with the enhancement in air temperature and IR power. The highest RR was observed at an air temperature of 70 °C and IR power of 750 W, as the increase in air temperature and IR power prevented tissue destruction and reduced product shrinkage due to the shorter drying time [65,66]. Similar results were observed during pomegranate drying using a hybrid CV/IR dryer by Briki et al. [67]. El-Mesery et al. [68] investigated the effect of a hybrid dryer (CV/IR) on the qualitative characteristics of garlic slices and found an increment in the RR with the increase in temperature and IR power which is consistent with the present research.

### 3.4. Bioactive Properties

#### 3.4.1. Total Phenol Content (TPC)

**Figure 6:**
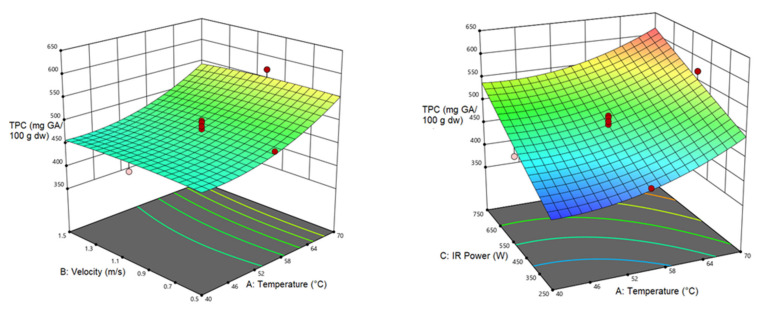

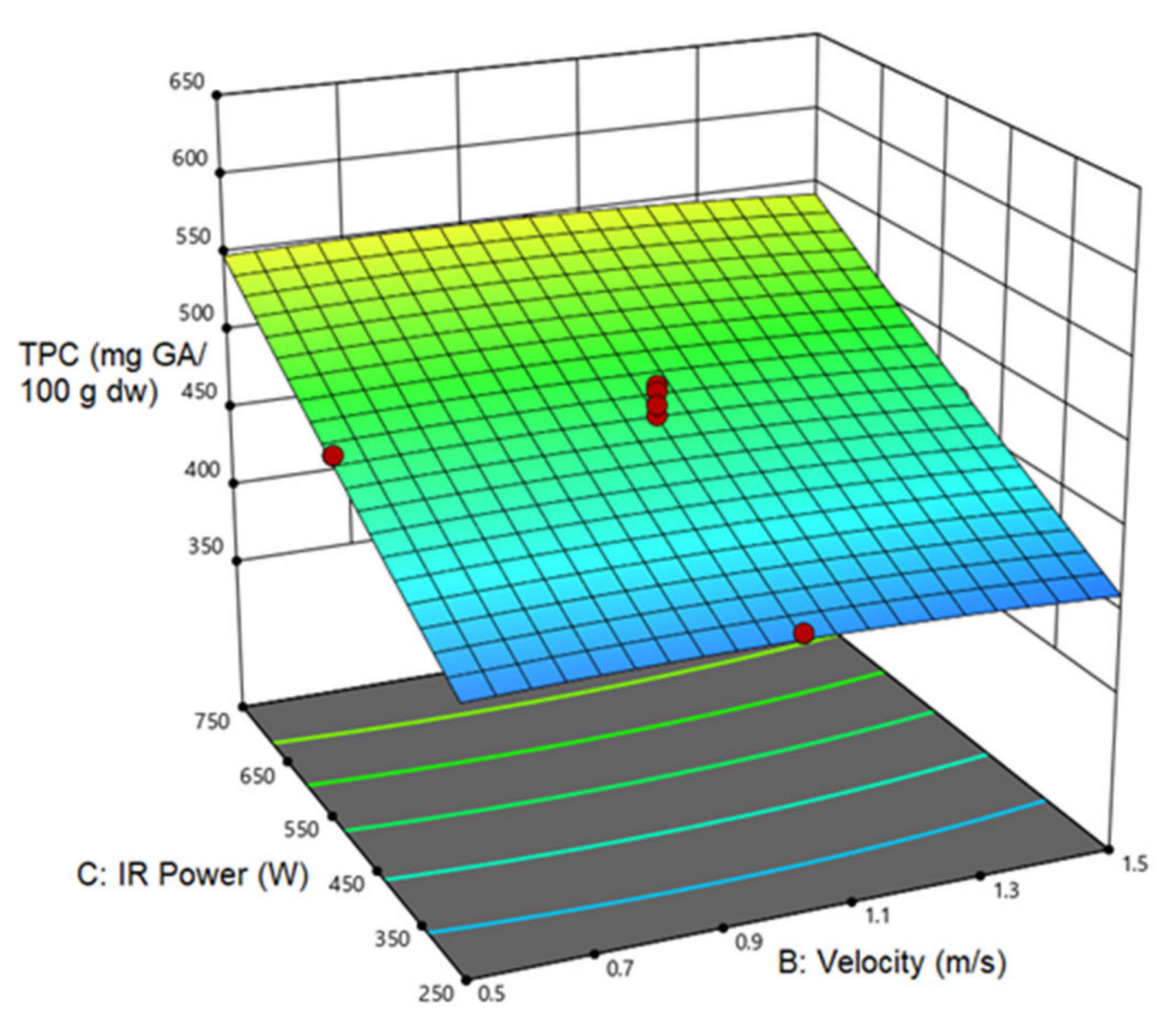
Interaction of air temperature/air velocity, air temperature/IR power, and IR power/air velocity on TPC of pumpkin.

According to Table 10, the analysis of variance for TPC shows a significant linear relationship between air temperature and IR power with TPC (*p* < 0.0001). The second-order expression of air temperature was also significant (*p* < 0.001). Figure 6 shows the effect of independent variables on TPC response. According to Figure 6, TPC increases with raising the air temperature, while the air velocity had no significant effect. Moreover, TPC exhibited a descending trend with declining IR power and air temperature. The reduction in TPC during the drying process can be assigned to the irreversible oxidative reactions and thermal decomposition during long-term heating [69]. Examining TPC in samples dried by the CV/IR method compared to those dried by the hot air and IR method suggested higher levels of TPC. In the CV/IR method, bioactive compounds are released due to changes in the structure. These changes can have adverse effects in terms of preserving bioactive compounds, but in this study, it showed a positive effect (increase) in TPC [42]. These findings agree with the reports of Vega-Galvez et al. [70] for drying papaya.

#### 3.4.2. Antioxidant Activity (*AA*)

**Figure 7:**
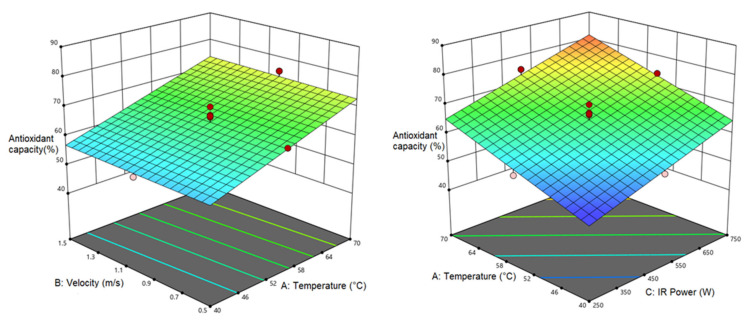
Interaction of air temperature/air velocity and air temperature/IR power on *AA* of pumpkin.

The highest and lowest *AA* contents in pumpkin samples were 83.24% (at 70 °C, 0.5 m/s, and 750 W) and 49.92% (at 40 °C, 0.5 m/s, and 250 W), respectively. Table 11 presents the analysis of variance for *AA* response. As seen, air temperature and IR power have a significant linear relationship (*p* < 0.0001) with *AA*. Figure 7 shows the effect of air temperature and air velocity on the *AA* in which the *AA* rose with the elevation in the air temperature. The air velocity, however, is not significant as it follows a constant trend. Concerning the effect of air temperature and IR power on *AA* response, the increase in both factors incremented the *AA* as the maximum *AA* was observed at 70 °C and 750 W, which could be due to the formation of a Maillard reaction [71]. Disturbance in the cell wall can result in the release of oxidizing and hydrolytic enzymes which can lead to the destruction of antioxidants in fruits and vegetables. The thermal process at low-temperatures, however, activates these enzymes and causes the loss of phenolic acid due to long-term exposure to heat. The increase in temperature, though, prevents the loss of phenolic acid due to less disturbance in the cell wall, and as a result, the antioxidant capacity increases [72]. Many other researchers also reported a rise in the *AA* with the enhancement in the temperature and IR power [22,59,73].

### 3.5. Vitamin C (VC)

**Figure 8:**
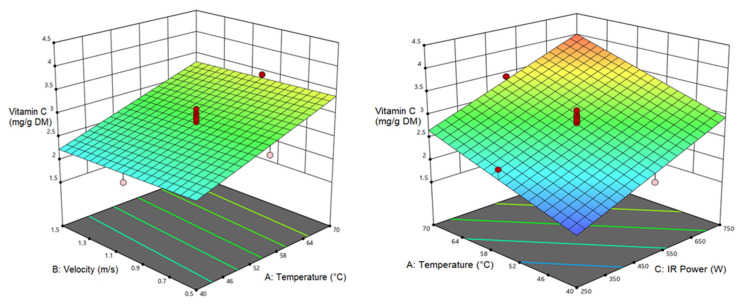
Interaction of air temperature/air velocity and air temperature/IR power on VC of pumpkin.

Vitamin C is a vital nutrient for human health. Thermal instability explains the selection of VC as an indicator of the heat treatment process. In general, preservation of VC could indicate that other nutrients are also preserved [74]. The VC content of pumpkin samples was in the range of 1.57 mg/g dw (temperature of 40 °C, air velocity of 1.5 m/s, and IR power of 250 W) which rose to 4.11 mg/g dw (at 70 °C, air velocity of 0.5 m/s, and IR power of 750 W). Vitamin C is highly sensitive to heat and will be rapidly oxidized. It will be decomposed by enhancing the drying time and temperature of the drying process, therefore, high-temperature and short-period drying approaches are recommended to prevent the loss of VC while achieving high drying efficiencies. In this way, moisture will be declined to a desirable level while avoiding VC loss. According to Table 12 for the ANOVA results of the VC response, there is a linear relationship between air temperature and IR power with VC (*p* < 0.0001); meanwhile, the air velocity showed no significant relationship. Figure 8 demonstrates the influence of the independent variables of the VC content; as seen, a rise in the air temperature and IR power enhanced the VC content. The minimum VC was observed at the lowest air temperature and IR power. Decline in VC can be assigned to the longer exposure of the samples in the drying chamber and increased IR intensity, which finally altered the nature of the product and resulted in its thermal damage. Previous results also mentioned the usefulness of the shorter drying times in preservation of VC [75,76].

### 3.6. Optimization

Optimal operation conditions were explored by numerical optimization techniques for the optimal drying of pumpkin slices using a CV/IR dryer. Here, the aim of optimization (see Table 13) is to minimize the drying time, SEC, shrinkage, and color variation in the pumpkin samples while maximizing their RR and TPC, VC, and AA. Table 13 presents the optimized independent and dependent variables for drying pumpkin slices by a CV/IR dryer. Accordingly, the optimal drying time involved the air temperature, air velocity, and IR power of 70 °C, 0.69 m/s, and 750 W, respectively. The mentioned optimal condition led to drying time, SEC, shrinkage, color variation, RR, TPC, *AA*, and VC of 72.53 min, 24.52 MJ/kg, 23%, 14.74, 4.97, 617.67 mg GA/100 g dw, 81.57%, and 4.02 mg/g dw, respectively, with a confidence level of 0.948. Table 14 lists the fitted model coefficients of the regression equation for the response variables (IR power, air velocity, and air temperature).

## 4. Conclusions

In the present research, effective parameters of the drying time, SEC, and physiochemical properties of pumpkin slices were assessed during drying by a thin film dryer using a RSM software and an axial central cubic design (CCD) considering three independent variables of IR power (250, 500, and 750 W) air temperature (40, 55, and 70 °C), and air velocity (0.5, 1 and 1.5 m/s) in a hybrid CV/IR dryer. The process time and specific energy consumption were decreased with increasing temperature and infrared power. Specific energy consumption for pumpkin drying is the lowest (24.52 MJ/kg) at drying temperature (70 °C) and IR power (750 W) and air velocity (0.5 m/s). The pumpkin slices dried at 70 °C and 750 W had the highest retention of VC, and antioxidant activity and total phenol contents. The results can be included in the design of an optimal dryer. The results indicated that the presented models had proper efficiency in predicting, optimizing, and modifying the evaluated parameters during the drying process. The IR power and air temperature and speed were 750 W, 70 °C, and 0.69 m/s, respectively, which resulted in the desirability factor of 0.948. Application of the mentioned conditions can usefully decrease the waste and number of experiments for drying pumpkin slices using a hybrid CV/IR dryer.

## Figures and Tables

**Table 1:** Formulas used for determining the energy consumption of CV/IR.

Equation	Equation Number	References
$EU_{ter}=A\cdot \nu \cdot \rho_{a}\cdot C_{p}\cdot \Delta T\cdot t$	(2)	[41]
$EU_{mec}=\Delta P\cdot M_{air}\cdot t$	(3)	[42]
$EU_{CV}=EU_{ter}+EU_{mec}$	(4)	[43]
$EU_{IR}=K\cdot t\cdot 3600$	(5)	[44]
$E_{t(CV/IR)}=Equation\;(4)+Equation\;(5)$	(6)	[45]
$SEC=\left(\frac{E_{t}{}_{\left(CV/IR\right)}}{M_{W}}\right)$	(7)	[46]

**Table 2:** Independent variables and their levels.

Independent Variables	Coded Variables	Levels		
		−1	0	+1
Air temperature (°C)	X1	40	55	70
Air velocity (m/s)	X2	0.5	1	1.5
IR power	X3	250	500	750

**Table 3:** Experiment design and value of dependent variables at different levels of independent variables.

Number	Air Temperature (°C)	Air Velocity (m/s)	IR Power (W)	SEC (MJ/kg)	Drying Time (Min)	Shrinkage (%)	RR	Color	TPC (mg GA/100 g dw)	*AA* (%)	VC (mg/g dw)
1	40	1.5	750	64.74	130	31.29	2.79	20.29	540.35	63.69	3.11
2	55	1	500	45.54	130	33.34	3.24	22.11	488.88	66.89	2.89
3	55	1	250	51.71	170	40.40	2.22	26.65	420.24	54.56	2.35
4	55	1	500	44.32	120	31.25	3.2	20.25	468.65	70.11	2.64
5	40	0.5	750	29.92	170	34.49	2.66	22.22	539.19	60.68	2.96
6	55	1.5	500	58.8	120	35.59	3.26	23.35	466.69	62.69	2.45
7	55	1	500	42.11	125	32.23	3.44	21.29	501.21	67.25	2.83
8	70	0.5	750	24.52	75	22.54	4.98	14.25	611.29	83.24	4.11
9	55	0.5	500	29.26	140	34.57	3.11	24.23	486.34	64.57	2.65
10	40	1	500	54.04	180	42.24	2.29	25.22	444.35	55.11	2.08
11	55	1	750	41.91	95	29.29	4.22	18.23	545.55	73.80	3.19
12	55	1	500	46.54	130	35.56	2.95	23.05	455.88	66.52	2.94
13	70	0.5	250	32.31	120	29.45	2.56	25.2	485.65	60.61	2.67
14	55	1	500	48.25	135	38.87	3.15	22.04	482.50	62.50	3.01
15	55	1	500	50.50	140	33.14	3.33	21.9	497.25	60.99	3.11
16	70	1.5	250	57.83	100	34.59	2.77	26.01	480.57	64.59	2.44
17	70	1	500	39.53	80	28.87	3.76	20.21	570.30	75.25	3.42
18	70	1.5	750	39.32	60	25.11	4.66	15.01	605.59	82.25	4.08
19	40	0.5	250	49.19	250	49.95	1.75	32.07	388.96	49.92	1.64
20	40	1.5	250	80.36	230	47.89	1.82	30.22	393.34	51.59	1.57

**Table 4:** Statistical values fitted for dependent variables by response surface method.

Source	Drying Time	SEC	Shrinkage	Color	RR	TPC	AA	VC
Model (*p*-value)	0.0001 ^a^	0.0001 ^a^	0.0001 ^a^	0.0001 ^a^	0.0001 ^a^	0.0001 ^a^	0.0001 ^a^	0.0001 ^a^
Lack of Fit (*p*-value)	0.6159 ^ns^	0.5868 ^ns^	0.9985 ^ns^	0.3221 ^ns^	0.4103 ^ns^	0.8821 ^ns^	0.8137 ^ns^	0.3368 ^ns^
R^2^	0.9853	0.9628	0.9476	0.9415	0.9603	0.9585	0.9147	0.9248
Adj. R^2^	0.9801	0.9528	0.9337	0.9346	0.9528	0.9507	0.9047	0.916
Predicted R^2^	0.964	0.9183	0.9315	0.9208	0.9327	0.9399	0.8796	0.9031
C.V.	4.96	6.12	5.07	4.9	5.89	2.76	4.27	6.73
Std. Dev.	6.69	2.85	1.75	1.11	0.18	13.61	2.77	0.19

^a^ = significant at 0.1%. ^ns^ = Not significant.

**Table 5:** Analysis of variance for drying time response using RSM.

Source	Sum of Squares	df	Mean Square	F Value	*p*-Value Prob > F	
Model	42,073.13	5	8414.63	187.92	<0.0001	significant
A-Air temperature	27,562.50	1	27,562.50	615.55	<0.0001	
B-Velocity	1322.50	1	1322.50	29.54	<0.0001	
C-IR power	11,560.00	1	11,560.00	258.17	<0.0001	
AC	1128.13	1	1128.13	25.19	0.0002	
C^2^	500.00	1	500.00	11.17	0.0048	
Residual	626.87	14	44.78			
Lack of Fit	376.87	9	41.87	0.84	0.6159	not significant
Pure Error	250.00	5	50.00			
Cor Total	42,700.00	19				

**Table 6:** Analysis of variance for SEC response using RSM.

Source	Sum of Squares	df	Mean Square	F Value	*p*-Value Prob > F	
Model	3150.12	4	787.53	96.97	<0.0001	significant
A-Air Temperature	717.82	1	717.82	88.38	<0.0001	
B-Velocity	1845.92	1	1845.92	227.29	<0.0001	
C-IR power	503.96	1	503.96	62.05	<0.0001	
AB	82.43	1	82.43	10.15	0.0061	
Residual	121.82	15	8.12			
Lack of Fit	78.32	10	7.83	0.90	0.5868	not significant
Pure Error	43.50	5	8.70			
Cor Total	3271.95	19				

F-value: The F-value in the ANOVA also deteremins the *p*-value.

**Table 7:** Analysis of variance for color response using RSM.

Source	Sum of Squares	df	Mean Square	F Value	*p*-Value Prob > F	
Model	337.59	2	168.79	136.79	<0.0001	significant
A-Air Temperature	86.08	1	86.08	69.76	<0.0001	
C-IR power	251.50	1	251.50	203.82	<0.0001	
Residual	20.98	17	1.23			
Lack of Fit	16.59	12	1.38	1.58	0.3221	not significant
Pure Error	4.38	5	0.88			
Cor Total	358.56	19				

**Table 8:** Analysis of variance for shrinkage response using RSM.

Source	Sum of Squares	df	Mean Square	F Value	*p*-Value Prob > F	
Model	832.87	4	208.22	67.88	<0.0001	significant
A-Air Temperature	426.41	1	426.41	139.00	<0.0001	
C-IR power	354.74	1	354.74	115.64	<0.0001	
AB	21.03	1	21.03	6.85	0.0194	
AC	30.69	1	30.69	10.01	0.0064	
Residual	46.01	15	3.07			
Lack of Fit	8.02	10	0.80	0.11	0.9985	not significant
Pure Error	38.00	5	7.60			
Cor Total	878.88	19				

**Table 9:** Analysis of variance for RR response using RSM.

Source	Sum of Squares	df	Mean Square	F Value	*p*-Value Prob > F	
Model	12.95	3	4.32	128.93	<0.0001	significant
A-Air temperature	5.51	1	5.51	164.42	<0.0001	
C-IR power	6.71	1	6.71	200.32	<0.0001	
AC	0.74	1	0.74	22.04	0.0002	
Residual	0.54	16	0.033			
Lack of Fit	0.40	11	0.036	1.30	0.4103	not significant
Pure Error	0.14	5	0.028			
Cor Total	13.49	19				

**Table 10:** Analysis of variance for TPC response using RSM.

Source	Sum of Squares	df	Mean Square	F Value	*p*-Value Prob > F	
Model	68,356.50	3	22,785.50	123.06	<0.0001	significant
A-Air temperature	19,999.68	1	19,999.68	108.01	<0.0001	
C-IR power	45,321.17	1	45,321.17	244.77	<0.0001	
A^2^	3035.65	1	3035.65	16.39	0.0009	
Residual	2962.58	16	185.16			
Lack of Fit	1453.87	11	132.17	0.44	0.8821	not significant
Pure Error	1508.71	5	301.74			
Cor Total	71,319.08	19				

**Table 11:** Analysis of variance for *AA* response using RSM.

Source	Sum of Squares	df	Mean Square	F Value	*p*-Value Prob > F	
Model	1400.46	2	700.23	91.18	<0.0001	significant
A-Air temperature	721.65	1	721.65	93.97	<0.0001	
C-IR power	678.81	1	678.81	88.39	<0.0001	
Residual	130.56	17	7.68			
Lack of Fit	74.20	12	6.18	0.55	0.8173	not significant
Pure Error	56.36	5	11.27			
Cor Total	1531.02	19				

**Table 12:** Analysis of variance for TPC response using RSM.

Source	Sum of Squares	df	Mean Square	F Value	*p*-Value Prob > F	
Model	7.47	2	3.73	104.56	<0.0001	significant
A-Air Temperature	2.87	1	2.87	80.43	<0.0001	
C-IR power	4.60	1	4.60	128.70	<0.0001	
Residual	0.61	17	0.036			
Lack of Fit	0.48	12	0.040	1.52	0.3368	not significant
Pure Error	0.13	5	0.026			
Cor Total	8.08	19				

**Table 13:** The results of optimizing the process of drying pumpkin samples with CV/IR.

IR Power (W)	Air Velocity (m/s)	Air Temperature (°C)	Drying Time (Min)	SEC (MJ/kg)	Color	Shrinkage (%)	RR	TPC (mg GA/100 g dw)	*AA* (%)	VC (mg/g dw)
750	0.69	70	72.53	24.52	14.74	23	4.97	617.97	81.57	4.02

**Table 14:** Coefficients of the model fitted to the regression equation of response variables (A, air temperature, B, air velocity, and C, IR power).

Response	Intercept	A	B	C	AB	AC	BC	A^2^	C^2^
SEC	46.53	−8.472 ^a^	13.586 ^a^	−7.099 ^a^	−3.209 ^a^				
Drying time	130	−52.5 ^a^	−11.5 ^a^	−34 ^a^		11.875 ^a^			10 ^a^
Shrinkage	34.53	−6.53 ^a^		−5.956 ^a^	1.621 ^b^	1.958 ^a^			
Rehydration ratio	3.108	0.742 ^a^		0.819 ^a^		0.303 ^a^			
Color	22.69	−2.934 ^a^		−5.015 ^a^					
TPC	481.319	44.721 ^a^		67.321 ^a^				24.64 ^a^	
Antioxidant capacity	64.840	8.495 ^a^		8.239 ^a^					
Vitamin C	2.807	0.536 ^a^		0.678 ^a^					

^a^ = significant at 0.01%; ^b^ = significant at 0.05%.

## Data Availability

The data presented in this study are available on request from the corresponding author.

## Nomenclature

*AA*	%	Antioxidant activity
*A*	m^2^	Tray area
*A_S_*	-	Absorbance values of the blank
*A_t_*	-	Absorbance values of the sample
$C_{p}$	kJ/kg °C	Specific heat
$E_{t}$	kJ	Energy consumption
$EU_{mec}$	kJ	Mechanical energy
$EU_{IR}$	kJ	Energy consumption in infrared dryer
$EU_{CV}$	kJ	Energy consumption in convective dryer
$EU_{ter}$	kJ	Thermal energy consumption
*K*	W	IR power (W)
*MC_d.b_*	% d.b	Moisture content dry basis
$M_{W}$	kg	Weight loss
$S_{a}$	%	Shrinkage
$SEC_{CV/IR}$	kJ/kg	Energy consumption in CV/IR dryer
*V_0_*	cm^3^	Initial volume
*V_t_*	cm^3^	Final volume
*υ*	m/s	Inlet air velocity
*t*	s	Drying time
$W_{r}$	kg	Weight of the sample before rehydration
$W_{d}$	kg	Weight of the sample after rehydration
*Wi*	kg	Initial weight of the samples
*Wd*	kg	Dry matter
$\rho_{a}$	kg/m^3^	Air density
Δ*T*	°C	Temperature difference
Δ*P*	mbar	Pressure difference
Δ*L**, Δ*b**, Δ*a**		The difference between the color of fresh and dried samples
